# Community physicians’ knowledge of secondary prevention after ischemic stroke: a questionnaire survey in Shanxi Province, China

**DOI:** 10.1186/s12909-015-0481-4

**Published:** 2015-11-03

**Authors:** Chen Chen, Xiaoyuan Qiao, Huijie Kang, Ling Ding, Lixia Bai, Jintao Wang

**Affiliations:** 1 Department of Epidemiology, School of Public Health, Shanxi Medical University, Taiyuan, Shanxi Province China; 2 Department of Geriatrics, The Third People’s Hospital of Shanxi Province, Taiyuan, Shanxi China

**Keywords:** Ischemic stroke, Transient ischemic attack, Secondary prevention, Community physician

## Abstract

**Background:**

This cross-sectional, questionnaire-based survey, conducted in Shanxi Province, China, evaluated the knowledge of community physicians of secondary prevention of ischemic stroke and transient ischemic attacks (TIAs).

**Methods:**

A total of 1910 physicians practicing at 832 community-based clinics, hospitals and other care centers in 11 prefectures of Shanxi Province completed the questionnaires between 1 July and 30 September 2013.

**Results:**

Over 90 % of participants were aware of the most common risk factors for stroke, but lifestyle-related factors were seen as of low or medium importance for secondary prevention. Only about 50 % of physicians were aware of the existence of commonly used stroke scales, and fewer said that they would use those scales in their clinical practice. There were slight differences in the responses to some of the questions on risk factors and stroke scales were associated with the physicians’ gender, academic qualifications, practice duration and location. Less than half of the participants were aware of the secondary prevention recommendations included in the most recent guidelines.

**Conclusion:**

The survey revealed a huge gap in knowledge of current guidelines for secondary prevention of ischemic stroke and TIA among the physicians surveyed. Continuing education and training of community physicians, administered as a public health program, is needed to improve the healthcare of ischemic stroke and TIA patients.

## Background

The mortality of ischemic and hemorrhagic stroke is decreasing worldwide, resulting in an increase in the number of stroke survivors, especially in low- and middle-income countries [1]. Patients who have had an ischemic stroke or transient ischemic attack (TIA) have a high risk of recurrence; and in Western countries, approximately 8–12 % of patients experience a second event within the first year after a stroke or TIA [2, 3]. The National Stroke Registry of China reported that 17.7 % of 11,560 patients with ischemic stroke or TIA, experienced a recurrence within 1 year [4]. This geographical difference may be partly explained by the relatively high prevalence of intracranial large-artery disease in the Chinese compared with Western populations and the particularly high risk of recurrent ischemic stroke with that etiology. On the other hand, it might also be attributed to gaps in the professional education of practicing physicians and their familiarity with current guidelines for secondary prevention in China [5]. Such gaps would hinder timely and accurate risk evaluation, stratification, and optimal medical management, as well as the best use of antiplatelet, anticoagulant, or interventional/surgical therapies, which are all important for secondary prevention. Improved knowledge and awareness would be expected to result in a reduced risk of recurrence in stroke patients [6, 7].

**Fig. 1:**
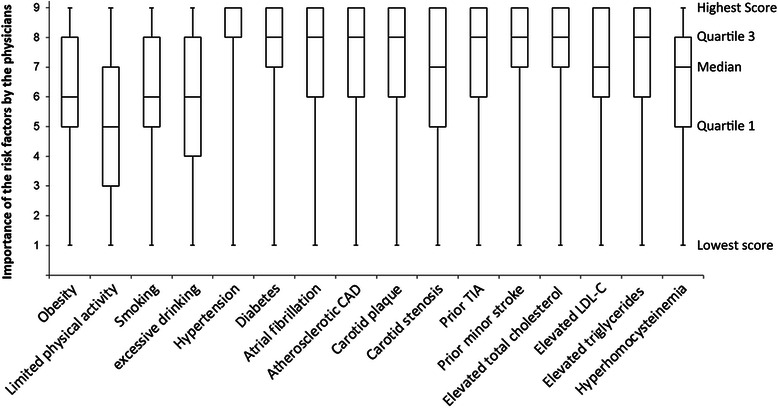
Box-and-whisker plots showing the importance of individual risk factors of ischemic stroke as rated on a scale from 1 to 9 by community physicians, with 9 as most important. CAD, coronary artery disease; TIA, transient ischemic attack; LDL-C, low-density lipoprotein cholesterol

A large, nationwide survey of secondary stroke prevention, conducted in 3489 Chinese general physicians and neurologists, reported a lack of adequate awareness of the Chinese National Guidelines for Prevention of Ischemic Stroke and Transient Ischemic Attack [8] The survey participants were either general practitioners or specialists [5], but their responses did not accurately represent the level of professional knowledge that many practicing Chinese community physicians have. For example, in Shanxi Province, located in the center of the Yellow River Valley of northern China, all community physicians are involved in the care of stroke patients. However, little is known of their knowledge of the aspects of stroke relevant to recurrence, and no standardized training or continuing education programs in the area of stroke are currently available to them. This survey of the community physicians in Shanxi Province, where our medical university is located, was conducted to identify existing gaps in the professional knowledge of stroke. The results may assist in the implementation of appropriate continuing education programs intended to reduce the risk of recurrent stroke.

## Methods

### Ethics statement

This cross-sectional study surveyed currently licensed physicians practicing in community-based clinics, hospitals and other care centers in Shanxi Province, China, and was approved by the Ethics Committee of Shanxi Medical University. A questionnaire with selected response questions was mailed to all physicians practicing in clinics in the 11 prefectures of Shanxi Province, and all of those who responded provided written, informed consent before completing the survey.

### Questionnaire survey

The Shanxi Provincial Health Bureau sent the questionnaires to the local health authorities of 11 prefectures in the province, which then forwarded them to all the general physicians in their communities. A pilot survey of 10 physicians in two communities was conducted before initiation of the study to estimate the response and questionnaire completion rates of potential participants.

The participants were asked to provide personal information including their age, gender, and demographic information, including the number of years in practice and their academic qualifications. Questions on knowledge of secondary prevention of ischemic stroke and TIA were developed to reflect the most recent Chinese stroke rehabilitation management [9] and American Heart Association/American Stroke Association (AHA/ASA) guidelines [7, 10]. The questionnaire covered three major areas. The first part consisted of multiple-choice questions about common risk factors of ischemic stroke  is shown in Fig. 1, including obesity, limited physical activity, smoking, excessive drinking, histories of hypertension, diabetes, atrial fibrillation, atherosclerotic coronary/carotid artery diseases, prior TIA/minor stroke, dyslipidemia (elevated total cholesterol, low-density lipoprotein cholesterol, or triglycerides) and hyperhomocysteinemia [7, 10]. The participants were asked for their awareness of these risk factors, and to rate each risk factor on a scale of 1 to 9, with 9 indicating the highest importance. The second part consisted of “yes or no” questions related to the awareness of commonly used clinical scores to evaluate the severity or prognosis of ischemic stroke or TIA. The questions were based on the National Institutes of Health Stroke Scale (NIHSS) [11] and its Chinese version (C-NIHSS) [12], and the Scandinavian Stroke Scale (SSS) [13], as well as the ABCD score (A, age; B, blood pressure; C, clinical features; and D, duration of symptoms) for TIA [14]. The physicians were also asked if they would use those scores to evaluate any suspected ischemic stroke or TIA cases. The third part examined knowledge of acute treatment and secondary prevention of ischemic stroke, and consisted of multiple-choice questions on aspects such as thrombolytic, antiplatelet, anticoagulant, and antihypertensive therapies.

### Statistical analysis

Descriptive statistics were used to summarize the study results. Age and number of years in practice were expressed as means ± SD. Ordinal variables (e.g., scoring of the importance of risk factors) were expressed as medians with interquartile (IQR) ranges and box-and-whisker plots. Categorical demographic variables and responses to the multiple-choice and “yes or no” items in the questionnaire were given as numbers (percentages). The number of community physicians who completed and returned the questionnaire was considered as the denominator for all proportion calculations. Bar charts were used to reveal the community physicians’ awareness of common risk factors and scoring systems for ischemic stroke or TIA. Chi-square tests were performed to determine the significance of differences in the awareness of risk factors and stroke scales in male and female physicians and in physicians with different academic qualifications, practice durations, and practice locations. All statistical analyses were carried out by SPSS version 13.0 (SSPS Inc, Chicago, USA). Two-sided *P*-values <0.05 were considered statistically significant.

## Results

### Participant demographics

A total of 1,918 questionnaires were mailed to physicians at 832 community-based clinics, hospitals and other care centers in 11 prefectures of Shanxi Province, China, from 1 July to 30 September 2013. Of these, 1,910 were completed and returned, for a response rate of over 99 %. Of the participating physicians, 780 (40.8 %) were men, and 1130 (59.2 %) were women. Their mean age was 43 ± 14 years, and they had practiced for a mean of 15 ± 15 years. The highest academic qualification in this group of community physicians was a 3-year junior college degree in medicine, earned by 1346 participants (70.5 %), 455 (23.8 %) had a 4-year bachelor’s degree, and 15 (0.8 %) had an additional 2 to 3-year master’s degree. Ninety-four participants (4.9 %) were traditional Chinese medicine practitioners who had been trained by their predecessors and did not have a formal tertiary qualification.

### Knowledge of common risk factors of ischemic stroke

Awareness of common risk factors for ischemic stroke, as measured by the questionnaire, is shown in Table 1. More than 90 % the surveyed physicians were aware of the risk factors. However, nearly 30 % did not consider limited physical activity as a stroke risk; and obesity, smoking, and excessive alcohol consumption were under appreciated, as indicated by median scores of 5 or 6 on a scale in which 9 indicated the highest importance.

**Table 1:** Gender, academic qualifications, practice duration and location and awareness of common risk factors for ischemic stroke^a^

	Total	Gender			Highest academic qualification					Practice duration				Location of practicing		
	N = 1910	Male	Female	P value^*^	Junior college degree	Bachelor’s degree	Master’s degree	TCM practitioners with no college education (n = 94)	P value^*^	<10 years	10–20 years	>20 years	P value^*^	Rural	Urban	P value^*^
		(n = 780)	(n = 1,130)		(n = 1,346)	(n = 455)	(n = 15)			(n = 650)	(n = 917)	(n = 343)		(n = 454)	(n = 1,456)	
Obesity	96.7	96.6	96.9	0.807	96.5	96.8	100	100	0.321	96.5	96.4	97.4	0.614	98.0	96.4	0.154
Limited physical activity	73.2	73.3	73.0	0.884	74.1	67.6	66.7	76.8	0.070	76.3	73.2	68.4	0.037	74.9	72.7	0.431
Smoking	96.3	96.6	96.0	0.475	96.1	95.5	100	98.8	0.457	96.9	95.8	96.7	0.561	97.4	96.0	0.219
Excessive drinking	94.3	93.2	95.0	0.123	93.5	95.8	93.3	98.8	0.108	95.5	94.7	92.0	0.069	93.0	94.6	0.239
Hypertension	99.7	99.6	99.8	0.402	99.7	99.8	100	100	0.969	99.8	99.5	99.8	0.738	99.7	99.7	0.982
Diabetes	96.1	96.7	95.7	0.321	95.6	97.3	86.7	96.4	0.137	95.8	96.4	95.3	0.669	92.5	97.0	<0.001
Atrial fibrillation	92.0	90.8	92.7	0.152	91.5	93.5	93.3	87.7	0.308	89.5	91.9	92.4	0.278	89.1	92.7	0.028
Atherosclerotic CAD	95.2	93.6	96.2	0.013	95.1	94.8	93.3	96.3	0.930	92.7	96.5	93.6	0.930	94.7	95.3	0.648
Carotid plaque	96.7	95.9	97.2	0.146	95.9	98.2	100	100	0.032	95.2	96.4	97.6	0.170	96.5	96.7	0.841
Carotid stenosis	94.9	94.3	95.2	0.408	93.9	96.7	93.3	97.5	0.115	92.6	95.7	94.5	0.090	92.6	95.4	0.037
Prior TIA	98.0	97.2	98.5	0.056	97.5	99.5	93.3	96.4	0.041	98.1	97.9	97.2	0.616	98.2	97.9	0.720
Prior minor stroke	98.0	97.7	98.1	0.545	97.9	98.0	93.3	97.5	0.665	97.6	97.6	98.1	0.848	99.4	97.6	0.038
Elevated total cholesterol	98.3	97.9	98.5	0.462	97.5	99.8	100	100	0.015	97.9	98.1	98.6	0.715	99.1	98.0	0.163
Elevated LDL-C	92.2	92.4	92.1	0.927	91.8	94.1	80.0	89.0	0.092	93.5	91.6	89.5	0.121	91.8	92.3	0.761
Elevated triglycerides	98.0	97.8	98.2	0.604	97.3	99.5	100	100	0.021	97.6	97.8	98.1	0.872	99.1	97.8	0.100
Hyperhomocysteinemia	89.9	88.4	90.9	0.094	89.1	92.2	80.0	86.8	0.149	91.1	90.6	84.7	0.004	87.9	90.4	0.182

*TCM* traditional Chinese medicine, *CAD* coronary artery disease, *TIA* transient ischemic attack, *LDL-C* low-density lipoprotein cholesterol

^*^Chi-square test, *P* < 0.05 indicates a significant difference in awareness between groups.

^a^Values are given as percentages

Differences in the perceived importance of individual stroke-risk factors that were reported by male and female physicians and by physicians with different academic qualifications, duration and location of practice are also shown in Table 1. There was no significant difference between male and female physicians in awareness of the surveyed risk factors, except for atherosclerotic coronary artery disease, which female physicians scored as being more important than male physicians did (*P* = 0.013). Physicians with shorter practice durations were more aware of the importance of limited physical activity and hyperhomocysteinemia as risk factors than were physicians in practice for longer periods of time. The degree of awareness of a number of risk factors were significantly different in community physicians with different academic qualifications or practice location, but the absolute differences in percentage were small and may not be meaningful in practice.

### Knowledge of commonly used stroke scales

Awareness of commonly used stroke and TIA evaluation scores and their willingness to use them in their practices are shown in Table 2. Only 39.8 %, 55.0 % and 20.0 % of the community physicians, respectively, were aware of using the NIHSS, C-NIHSS, and SSS scores to evaluate the neurological deficits of stroke or TIA patients, and only 45.4 % of the physicians indicated they would use NIHSS or C-NIHSS criteria to evaluate potential stroke patients in their clinical practice. Only 46.9 % of the physicians were aware of the ABCD score, and only 18.9 % of them would use it to evaluate potential TIA patients in their clinical practice.

**Table 2:** Gender, academic qualification, practice duration, and location and awareness of and willingness to use commonly used stroke-scoring scales^a^

	Total	Gender			Highest academic qualification					Practice duration				Location of practicing		
	N = 1910	Male	Female	P value*	Junior college degree	Bachelor’s degree	Master’s degree	TCM practitioners with no college education	P value*	<10 years	10–20 years	>20 years	P value*	Rural	Urban	P value*
		(n = 780)	(n = 1,130)		(n = 1,346)	(n = 455)	(n = 15)	(n = 94)		(n = 650)	(n = 917)	(n = 343)		(n = 454)	(n = 1,456)	
Awareness of NIHSS	39.8	42.2	38.4	0.120	38.3	43.0	50.0	32.0	0.171	37.8	38.7	40.9	00.643	36.8	40.7	0.192
Awareness of C-NIHSS	55.0	56.3	54.1	0.363	54.1	59.3	60.0	41.7	0.021	51.7	55.9	53.7	0.370	47.5	56.9	0.001
Use of NIHSS/C-NIHSS in stroke cases	45.4	46.0	45.0	0.721	46.3	41.7	50	41.1	0.390	41.8	45.7	50.0	0.113	50.0	44.3	0.098
Awareness of SSS	20.0	21.4	19.2	0.244	18.0	24.5	13.3	19.0	0.033	18.9	16.1	22.1	0.041	21.6	19.6	0.404
Awareness of ABCD score	46.9	46.3	47.4	0.640	45.1	51.4	33.3	39.2	0.052	47.5	45.8	45.2	0.770	43.0	48.0	0.085
Use of ABCD score in TIA cases	18.9	19.8	18.4	0.730	18.8	17.2	23.1	13.3	<0.001	17.8	18.0	19.7	0.550	18.7	19.0	0.072

*TCM* traditional Chinese medicine, *NIHSS* National Institutes of Health Stroke Scale, *C-NIHSS* Chinese version of NIHSS, *SSS* Scandinavian Stroke Scale, ABCD acronyms: *A* Age, *B* Blood pressure, *C* Clinical features, *D* Duration of symptoms, *TIA* transient ischemic attack

^*^Chi-square test

^a^Values are reported as percentages

There was no significant difference between male and female physicians in their awareness of or willingness to use these scales (Table 2). The awareness of SSS and C-NIHSS differed among physicians with different practice duration and location as well as in those with the highest academic qualification and traditional Chinese medicine practitioners. There was a trend toward increased awareness of, and willingness to use, the NIHSS/C-NIHSS among those with bachelor’s or master’s degrees than among those without a tertiary qualification. Physicians with a junior college or a bachelor’s degree had more knowledge of the ABCD score than those with a master’s degree.

### Knowledge of secondary prevention strategies in ischemic stroke or TIA

The responses to questions on acute treatment and secondary prevention of ischemic stroke or TIA are shown in Table 3. Only 14.4 % of the community physicians were aware of the most recently recommended 4.5-h time window for intravenous thrombolytic therapy of acute ischemic stroke (Q1), while 35.9 % chose the 3-h time window that was included in the previous international guidelines [15]. Only around 50 % of the physicians were aware of the recommendations for antihypertensive therapy in ischemic stroke or TIA patients with elevated blood pressure (Q2, Q3) contained in the most recent guideline [7]. A total of 65.3 % of the physicians chose oral anticoagulants as the first-choice medication for cardioembolic ischemic stroke or TIA patients with atrial fibrillation (Q5), and 78.9 % were aware of the target range of the international normalized ratio (INR, Q6). Only 26.4 % were aware that the guidelines recommend the use of aspirin alone for patients who are unable to take oral anticoagulants (Q7) [7], and over half (52.8 %) chose clopidogrel plus aspirin. A majority of physicians (67.0 %) would use antiplatelet medications to prevent recurrence of noncardioembolic ischemic stroke, TIA, or other cardiovascular events (Q10). Only 40.5 % were aware that dual antiplatelet therapy with aspirin plus clopidogrel (Q11) is not currently recommended for routine secondary prevention after ischemic stroke or TIA [7]. In addition, only 11.4 % were aware of the recommendation for reducing homocysteine level (Q12), and 46.8 % were aware of the recommendation for postmenopausal hormone therapy (Q13) in ischemic stroke or TIA patients.

**Table 3:** Numbers and proportions of community physicians’ (*N* = 1,910) answers to multiple-choice questions on acute treatment and secondary prevention of ischemic stroke or TIA^a^

Questions & Answers	Numbers	Proportions
Q1. For eligible ischemic stroke patients, what is the recommended time window for intravenous fibrinolytic therapy with rtPA?		
3 hours	686	35.9 %
4 hours	180	9.4 %
4.5 hours^b^	275	14.4 %
6 hours	769	40.3 %
Q2. In secondary prevention for ischemic stroke or TIA, which of the following antihypertensive recommendations should be used in patients with elevated blood pressure?		
20/15 mmHg	330	17.3 %
15/10 mmHg	600	31.4 %
10/5 mmHg^b^	945	49.5 %
5/0 mmHg	35	1.8 %
Q3. If necessary to prevent recurrent stroke or other vascular events in ischemic stroke or TIA patients, when is it recommended to initiate antihypertensive therapy?		
Immediately	344	18.0 %
Beyond 24 hours^b^	1,112	58.2 %
Beyond 1 week	260	13.6 %
Beyond 2 weeks	194	10.2 %
Q4. What is the approximate percentage of cardiogenic cerebral embolism in all ischemic strokes?		
5 %	319	16.7 %
10 %	386	20.2 %
15 %	405	21.2 %
20%^b^	800	41.9 %
Q5. For ischemic stroke or TIA patients with AF, what is the recommended first-choice medication, if no contraindication exists?		
Antiplatelet medication	524	27.4 %
Oral anticoagulant^b^	1,247	65.3 %
Defibrase	105	5.5 %
Others, i.e., traditional Chinese medicine	34	1.8 %
Q6. What is the target INR for ischemic stroke or TIA patients with AF receiving anticoagulation with a vitamin K antagonist?		
1–2	180	9.4 %
2–3^b^	1,507	78.9 %
3–4	195	10.2 %
4–5	28	1.5 %
Q7. For ischemic stroke or TIA patients with AF who are unable to take oral anticoagulants, what medication is recommended?		
Aspirin alone^b^	504	26.4 %
Clopidogrel alone	398	20.8 %
Combination of clopidogrel and aspirin	1,008	52.8 %
Do not use any other medications	0	0
Q8. For ischemic stroke or TIA with acute myocardial infarction complicated by left ventricular mural thrombus formation identified by echocardiography or other cardiac imaging techniques, for at least how long should the patients be treated with oral anticoagulation?		
3 months^b^	514	26.9 %
6 months	760	39.8 %
9 months	120	6.3 %
12 months	516	27.0 %
Q9. For ischemic stroke or TIA patients with rheumatic mitral valve disease, is long-term warfarin therapy recommended whether or not AF is present?		
Yes^b^	1,215	63.6 %
No	487	25.5 %
No idea	208	10.9 %
Q10. For patients with noncardioembolic ischemic stroke or TIA, what medications are recommended to reduce the risk of recurrent stroke and other cardiovascular events?		
Antiplatelets^b^	1,280	67.0 %
Oral anticoagulants	29	26.1 %
Traditional Chinese medicine	131	6.9 %
Q11. Is aspirin plus clopidogrel recommended for routine secondary prevention after ischemic stroke or TIA?		
Yes	886	46.4 %
No^b^	774	40.5 %
No idea	250	13.1 %
Q12. Is there evidence that reducing homocysteine levels prevents stroke recurrence in ischemic stroke or TIA patients?		
Yes	1,322	69.2 %
No^b^	217	11.4 %
No idea	371	19.4 %
Q13. Is postmenopausal hormone therapy recommended for women with ischemic stroke or TIA?		
Yes	562	29.4 %
No^b^	894	46.8 %
No idea	454	23.8 %

*AF* atrial fibrillation, *INR* international normalized ratio, *rtPA* recombinant tissue-type plasminogen activator, *TIA* transient ischemic attack

^a^The questions and answers were designed to reflect relevant American Heart Association/American Stroke Association guidelines (AHA/ASA) [7, 10]

^b^Recommendations from relevant AHA/ASA guidelines [7, 10]

## Discussion

We used a questionnaire to survey the knowledge of acute treatment and secondary prevention for ischemic stroke and TIA in a group of 1910 community physicians with an average of 15 years of practice in Shanxi Province, China. The responses revealed a huge gap among the participants in knowledge and clinical application of the most recent Chinese and AHA/ASA treatment guidelines for secondary prevention of ischemic stroke and TIA. The results demonstrate the need to implement a new policy for continuing medical education and training in advances in the care of stroke and TIA patients. Keeping up-to-date with new developments in patient care and secondary prevention would reduce the risk of stroke recurrence.

Most community physicians were aware of the common risk factors for ischemic stroke and TIA. However, despite the extensive evidence that lifestyle modifications have beneficial effects on multiple stroke risk factors, many of the physicians surveyed considered lifestyle-related risk factors to be less important than the other modifiable or nonmodifiable risk factors. Gender, academic level, practice duration, and practice location all affected physician awareness of some of the risk factors surveyed, but the differences were not significant. Knowledge of commonly used stroke scales was severely inadequate regardless of gender, academic qualifications, practice duration, or practice location. Approximately half the physicians were aware of NIHSS, C-NIHSS, SSS, and ABCD scores, but fewer than half were willing to use those scores to evaluate stroke or TIA patients. In particular, timely use of the ABCD score in TIA patients could help to identify those at high risk of a subsequent stroke in the first few hours and days after TIA onset [14]. Standard procedures stipulate that such patients should be promptly transferred or referred to a tertiary hospital with a stroke care center. However, insufficient knowledge about use of the ABCD score may severely hinder timely risk stratification of TIA patients, especially for those whose symptoms have resolved and who seek help from community physicians without immediately going to the tertiary hospitals with stroke care centers. The community physicians participating in this study did not possess adequate knowledge of the secondary prevention measures recommended by the latest guidelines. Long-term adherence to these measures is crucial for preventing recurrent stroke and other cardiovascular events in those patients with post-ischemic stroke or TIA [7].

A study of over 2000 ischemic stroke or TIA patients discharged from 106 hospitals participating in the AHA’s Get With The Guidelines stroke program in the United States between July 2006 and July 2008, found that 87.1 %, 68.2 %, and 87.9 %, respectively, continued to adhere to antiplatelet, anticoagulant (warfarin), and antihypertensive medications at 1 year after discharge [16]. Since then, the Get With The Guidelines stroke program has significantly enhanced the quality of care of stroke patients in the United States, including secondary prevention [17]. Although similar programs have been established in China, (e.g., the China National Stroke Registry and the National Center for Stroke Care Quality Control) [18, 19], there are still huge evidence-based practice gaps in the secondary prevention of ischemic stroke and TIA. The long-term adherence to secondary prevention measures in Chinese stroke patients is lower than that reported in the American studies, especially in the use of anticoagulant medications. A national clinical trial with over 3000 ischemic stroke or TIA patients discharged from 47 hospitals in China, reported that at 1-year post stroke, 81.1 %, 36.1 % and 68.6 % of the patients, respectively, adhered to the antiplatelet, anticoagulant, and antihypertensive medications prescribed at discharge.cs [20] Since community physicians often prescribe daily medications for patients who have had an ischemic stroke or TIA, more education and communication programs are needed to equip them with better knowledge in this area. Moreover, providing community physicians with ready-to-use tools such as portable reference cards outlining stroke scores and essential secondary prevention recommendations of current guidelines, may help to narrow the evidence-based practice gaps in community care centers.

The strengths of this study are that all the 11 prefectures of Shanxi Province were surveyed and that the response rate was high, mainly because the physicians, as government employees, viewed participation as mandatory. Use of a simplified questionnaire that did not cover all the essential secondary prevention measures for ischemic stroke or TIA patients (e.g., management of lipid profiles and diabetes) was a study limitation. In the near future, independently designed studies of this kind that are nationwide in scope, or incorporated into national stroke registry programs, would further describe the gaps between community physician practices and evidence-based guidelines. The results of those studies would help in the design, and initiation of relevant education and support programs in China.

## Conclusions

This study revealed a huge gap among community physicians in the knowledge and clinical practice of the recommendations contained in the most recent Chinese and AHA/ASA guidelines for secondary prevention of ischemic stroke and TIA. There is a great need for education and training of the community physicians in this area.
